# Making integration work

**DOI:** 10.1111/1475-6773.13575

**Published:** 2020-12-07

**Authors:** David Blumenthal

**Affiliations:** ^1^ The Commonwealth Fund New York USA

For every complicated problem, there is a simple solution, and it is likely wrong.

In the collection of papers in this issue, the complex problem is a health care enterprise that is too costly, inequitable, and deficient in quality. The simple solution is integrated health systems. The studies reported here suggest that these systems, at least in the forms and at the times studied here, are not—or not yet—a miracle cure.

The concept of integration—and the coordination of care it implies—is powerful.^1^ For patients, it raises hopes that the many hands of the health care system will not only know what the others are doing, but gently pass patients among them; that warm, knowledgeable, and compassionate hand‐offs will be the rule rather than the exception; and that patients and their families will not be left to wander unassisted through our fragmented health system.

For clinicians, integration creates opportunities to optimize care quickly and easily, to reduce administrative burden, and to increase professional satisfaction. For policy makers, integration signals the possibility of reducing unnecessary duplication of services, improving safety and quality, and cutting costs.

The early returns from this research, however, suggest that on the ground, practice is not conforming to theory. Despite glimmers of hope, integration is not yielding significant improvements in documentable quality and cost of care.^2^ Only one of the papers addresses equity of care, finding no dramatic effects.^3^ None address effects on institutional racism.

There are a number of possible reasons for this disappointing result. First, despite decades of work, our measures of quality are still quite basic and may not capture subtle improvements in the management of complex conditions, where integration and coordination might make the most difference.^4^, ^5^

Second, the studies reported here may be occurring too early in the history of the integration movement to capture its ultimate impact. Realizing the promise of integration takes time. It takes time to change the behaviors of front‐line clinicians—their referral preferences, and their comfort with new colleagues and with reporting and compensation arrangements.^6^ It also takes time to install and optimize new information systems that provide the glue that binds systems together. Newly integrated health systems usually employ multiple legacy electronic health records that must be made interoperable (difficult) or ripped out and replaced by a single EHR (difficult and costly).

There is another more sobering reason that integrated systems may not be yielding hoped for benefits. The uncomfortable truth is that, despite all the rhetoric about quality improvement and cost reduction, the trend toward integration is as much about the financial welfare of affected organizations as anything else. In the battle between providers and payers, market power is everything, and size yields power. The result is a feeding frenzy in which financially strong (mostly) hospitals gobble up weaker competitors and physician practices in an effort to create bulletproof juggernauts that insurers simply have to have in their networks.

It would be unfair to say that the leaders of these organizations do not care about the quality and efficiency benefits of integration. They do. But in the current fee for service world, their boards hold them accountable first and foremost for their volumes, margins, and growth rates. The work required to achieve true integration and coordination simply does not garner the same internal rewards. What is more, boards generally consist of laypersons with deep financial expertise who readily understand spreadsheets on revenue and expense and the thrill of merger and acquisition, but fade into the wood paneling when the white coats show up to talk quality of care. Having served on the boards of several integrated systems, and worked as a clinician and senior manager in another, I can offer anecdotal support for these observations.

But setting all these other explanations aside, there is still another reason why the practice of integration may not live up to its promise. Creating integrated health systems that function seamlessly to optimize care for patients is devilishly hard. It requires strong, consistent, talented, and courageous leadership to overcome the huge barriers to making integrated system work at the front lines where it matters.^7^ Payment systems must strongly reward the benefits of integration—and penalize its failure—to stiffen the spines of boards and leaders and generate the will to succeed within new, far‐flung organizations. Only then will those organizations take on the innumerable local struggles required to align internal incentive and to stamp out internal rivalries and opposition. Only then will it be possible to work the deep cultural changes among clinicians that are required. Despite the emergence of new payment arrangements since the passage of the Affordable Care Act, they are not powerful or prevalent enough to have created the pressures required to motivate effective integration of care within many integrated systems.^8^

The research implications of these observations are numerous. First, studies of integrated systems need to be repeated when the organizations are mature. As a corollary, studies need to account for time since formation (however that is defined).

Still another area of potential work concerns how governance and payment may affect whether integration realizes its conceptual promise. There is a need for more descriptive work on chains of accountability within increasingly large, complex, and geographically dispersed health systems.^9^ In particular, we need to understand which (if any) boards are in charge, whether they have the competencies necessary to judge and direct the performance of the organizations under them, what their members’ priorities are, how they assess management's performance, and how they compensate senior leaders. If the hypothesized clinical benefits of integration are not high on controlling boards’ lists of concerns, and not considered in senior management compensation, it is unlikely that the integration will succeed in achieving nonfinancial goals.

With regard to payment, an important variable is the proportion of a system's patient revenue generated from at‐risk payment arrangements. It is hard to imagine how the tough work of integration can succeed without downside risk looming over the heads of organizational leaders.

Another important variable is whether local private and/or public actors hold integrated systems accountable for the total costs of care provided their patients. Such accountability could stem from the influence of a motivated dominant private payer, or from regulatory or quasi‐regulatory influences from state governments. For integrated systems that span multiple markets and political jurisdictions, capturing these effects will be especially challenging and may require examining the independent performance of operating units.

Studies should also probe the effects of market variables, and especially the level of competition among both providers and payers in local areas.^10^ It is reasonable to hypothesize that competition may either encourage or inhibit effective integration. In theory, fierce competition should motivate health systems to integrate effectively so as to cut costs, reduce prices, and pry business away from competitors. But it could just as easily drive acquisitions designed to assure market dominance and thus extract higher prices without having to make tough internal reforms.

Finally, it is past time to assess comprehensively the effects of integration on disparities in access and outcomes for individuals of different race and ethnicity. All studies of financial and organizational reforms should include such a focus unless there is a compelling reason not to.

For those of us who have tracked over decades the repeated efforts to improve health system performance, unexpected and often frustrating results are the rule rather than the exception. There is always, however, something to be learned from these experiments. In this case, the lesson may be in part that integration is a tool that can work or not depending on the incentives and resulting motives of the humans that employ it. The easy part of integration is moving the pieces around on the chess board of local markets. The hard part—deep, careful, continuous improvement of front‐line processes of care—will occur only if incentives bearing down on integrated systems support it.

Perhaps the integration experience suggests a variation on the truism that began this commentary: *For every complex health care problem, there is a simple solution, and it will not work unless we really want it to.*
